# Impact of Deep Brain Stimulation on Daily Routine Driving Practice in Patients with Parkinson's Disease

**DOI:** 10.1155/2015/608961

**Published:** 2015-11-10

**Authors:** Carsten Buhmann, Eik Vettorazzi, Christian Oehlwein, Fred Rikkers, Monika Poetter-Nerger, Alessandro Gulberti, Christian Gerloff, Christian K. Moll, Wolfgang Hamel

**Affiliations:** ^1^Department of Neurology, University Medical Center Hamburg-Eppendorf, Martinistrasse 52, 20246 Hamburg, Germany; ^2^Department of Medical Biometry and Epidemiology, University Medical Center Hamburg-Eppendorf, Martinistrasse 52, 20246 Hamburg, Germany; ^3^Neurological Outpatient Clinic for Parkinson's Disease and Deep Brain Stimulation, Lasurstrasse 27, 07551 Gera, Germany; ^4^Department of Neurophysiology and Pathophysiology, University Medical Center Hamburg-Eppendorf, Martinistrasse 52, 20246 Hamburg, Germany; ^5^Department of Neurosurgery, University Medical Center Hamburg-Eppendorf, Martinistrasse 52, 20246 Hamburg, Germany

## Abstract

*Objective.* To determine the influence of deep brain stimulation (DBS) on daily routine driving behavior in Parkinson's disease (PD) patients. *Methods.* A cross-sectional questionnaire survey was done in 121 DBS-PD patients. The influences of patient characteristics and DBS on current driving and driving at time of surgery and the predictive value of the preoperative levodopa-test on postoperative driving were evaluated. *Results.* 50% of 110 driving-license holders currently drove. 63.0% rated themselves as safe drivers, 39.4% reported improvement, and 10.9% noted deterioration in driving after DBS surgery. Inactive drivers had quit driving mainly due to disease burden (90.9%). Active drivers were younger, more often males, and less impaired according to H&Y and MMSE, had surgery more recently, and reported more often overall benefit from DBS. H&Y “on” and UPDRS III “off” scores at time of surgery were lower in pre- and postoperative active than in inactive drivers. Tremor and akinesia were less frequent reasons to quit driving after than before DBS surgery. Postoperatively, 22.7% (10/44) of patients restarted and 10.6% (7/66) of patients discontinued driving, independently of H&Y stage. The preoperative levodopa-test was not predictive for the postoperative driving outcome. *Conclusion.* 50% of PD patients with DBS drive. DBS surgery changes daily routine driving behavior.

## 1. Introduction

Driving in Parkinson's disease (PD) has been investigated intensively [1–3]. Although deep brain stimulation (DBS) has become an established therapy in the treatment of advanced PD, there is no published data about daily driving practice in PD patients with DBS. We have recently shown that subthalamic (STN) DBS has a positive impact on driving performance of PD patients in a driving-simulator setting [4]. Hitherto it is unknown to which extent DBS influences patients routine driving. To this end, we employed a questionnaire survey to investigate the influence of disease-related patient characteristics and the effect of DBS therapy on present driving behavior and to assess the driving frequency in PD patients with DBS. Furthermore the immediate effect of DBS surgery and the predictive value of the preoperative levodopa-test on postoperative driving practice were determined retrospectively.

## 2. Subjects and Methods

A questionnaire-based, monocentric, and cross-sectional survey was performed. Written informed consent was obtained from all subjects. The study was approved by the local ethics committee of the Medical Council Hamburg (addendum to trial PV3557).

From January 2013 until April 2014 all PD patients with DBS visiting the outpatient clinic at the University Medical Center Hamburg-Eppendorf were asked to fill out a self-developed questionnaire (details are given in Appendix A; English translation of the German questionnaire). The questionnaire included questions regarding driving experience, self-estimation of driving safety, dependence on a car, impact of driving on quality of life (QoL), expectation in DBS therapy regarding driving ability, overall benefit from DBS surgery (sections (1)–(3), (6), and (8)-(9) of the questionnaire), or symptoms, being eventually responsible for discontinuing driving (sections (5) and (7)). Further questions concerned the direct and short-term effect of DBS surgery on driving behavior. Therefore we asked retrospectively for the driving behavior within 3 and 12 months before and within 3 and 12 month after DBS surgery (section (4)). The physician's part of the questionnaire included questions regarding patient data, disease stage according to Hoehn and Yahr (H&Y), disease duration, clinical phenotype (tremor-dominant, akinetic-rigid, or equivalent), surgical target, clinic of DBS surgery, application of microelectrode recordings, and awareness of the physician whether the patient is driving a car. The cognitive score of the mini mental state examination (MMSE) within 3 months before or after time of survey was taken from the medical reports.

Moreover, influence of age, disease duration, motor impairment, and MMSE at time of DBS surgery and the predictive value of the preoperative levodopa-test on the postoperative driving practice were evaluated. Therefore, we checked the medical patient reports for the clinical H&Y and MMSE “on” state and the motor part of the Unified Parkinson's Disease Scale (UPDRS III) in the “on” and “off” condition of the preoperatively performed levodopa-test.

Only complete data sets were used for analysis (except missing MMSE in one subject). Data are presented as means and standard deviations [SD] for continuous data and are compared using *t*-tests or as counts and percentages for categorical data and compared using chi-square tests. Spearman's rank correlation was used to examine association between UPDRS scores and status of being an active driver. Association between H&Y score and likelihood to drive was calculated by linear-by-linear association within chi-square test and Spearman's rank correlation. Because this study was exploratory, no correction of statistical significance for multiple testing was performed to avoid inflating type II errors and thus missing real differences [5, 6].

## 3. Results

### 3.1. Basic Patient Characteristics at Time of Survey

121 consecutively PD patients with DBS (65.3% males) were investigated. None of the patients refused to take part in the survey. 91.7% (*n* = 111) of patients owned a valid driving license. Data set was complete in 110 of 111 cases (68.2% males) and considered for further analysis. 93 of the 110 patients were operated on in our center.  Table 1 provides characteristics for all patients and separately for currently active and inactive drivers. Group differences between currently active and inactive drivers were calculated and *p* values are given.

Clinical phenotype was equivalent in 63.7%, akinetic-rigid in 24.5%, and tremor-dominant in 11.8%. Microelectrode-guided DBS surgery was done in at least 105 (95.5%) patients (unknown for 5 patients). 50% (*n* = 55) of all patients were active drivers. Mean age was 65.2 [9.4] years. The questionnaire was filled out on average 4.1 [3.2] years after surgery. The subthalamic nucleus (STN) was the surgical target in 93.6% (*n* = 103) of cases. The ventrolateral thalamic base (VIM) was targeted in 5.4% (*n* = 6) of the patients. The surgical target of 1 patient (operated elsewhere) was unknown. Overall, 92.7% of patients reported benefit from DBS surgery and 90.0% declared they would redo the operation.

### 3.2. Patient Characteristics and Driving Related Safety Aspects at Time of Survey

62.7% (*n* = 69) of the patients rated themselves as safe drivers. These were 12.7% (*n* = 14); more patients than currently were driving (*n* = 55). 39.1% reported improved driving abilities due to DBS; 10.9% noted a deterioration of their driving skills. Six drivers (5.5%) were involved in a car accident within the last 12 months. Five of them (83.3%) considered themselves as safe drivers. Thus 7.2% of all patients who considered themselves as safe drivers (5/69) had an accident but only 2.4% (1/41) who considered themselves as unsafe drivers. Subtype of disease, age, gender, disease duration, MMSE, and duration of DBS were not significantly different in the subgroups of patients being or not being (94.5%) involved into a car accident within the last year.

**Figure 1:**
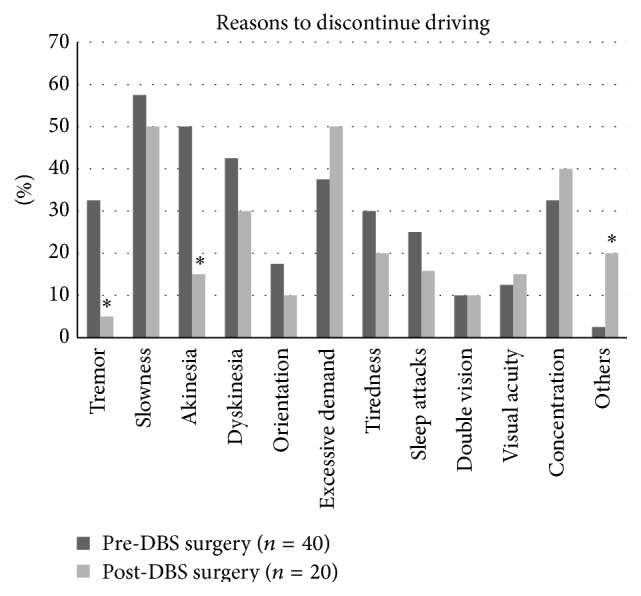
Parkinson's disease-related symptoms responsible for quitting driving. Symptoms related to Parkinson's disease and mentioned by the patients to be responsible alone or in combination for cessation of driving before or after DBS surgery are shown as occurrence in percentage (%). Significant different frequencies are labeled with an asterix (*∗*).

Forty-four patients had stopped driving prior to and 22 patients after brain surgery. The disease itself was reported to be the main reason for giving up driving (preoperatively in *n* = 40 [81.6%], postoperatively in *n* = 20 [90.9%] patients).  Figure 1 provides an overview of the different PD-related symptoms that forced patients to stop driving before and after DBS surgery until time of survey.

Only in 30.0% of cases the treating physician knew whether the patient was actually driving. 56 of the 66 (84.8%) preoperatively active drivers started to drive again within the first 3 months after DBS surgery.

### 3.3. Patient Characteristics of Active and Inactive Drivers at Time of Survey

Table 1 shows patient characteristics of all drivers, compares characteristics of currently active and inactive drivers, and gives an overview of the “status quo” of daily driving practice. Active drivers were significantly younger, less impaired according to H&Y and MMSE, and had shorter treatment duration with DBS compared to inactive drivers. Likelihood of driving a car decreases with increasing disease severity according to H&Y (*r* = −0.455, *p* < 0.001). Percentages of active drivers were 49.1% in H&Y 2, 43.6% in H&Y 3, 5.5% in H&Y 4, and 1.8% in H&Y 5.

Dependence on a car and reduced QoL without driving were reported significantly more often by currently active than inactive drivers. Active drivers more often reported benefit from DBS surgery and more often affirmed that they would undergo surgery again. 96.8% of the active drivers and 46.3% of the inactive drivers reported that they had expected to be able to drive after surgery.

Significantly more males than females were driving at time of survey (Table 1), and more males (69.0%) than females (48.3%) estimated themselves as safe drivers (*p* = 0.05). The expectation to be capable of driving after surgery was higher in males (78.2%) than in females (40.0%; *p* < 0.001).

PD patients, who quit driving postoperatively, less often reported tremor (5.0% [*n* = 1/20] versus 32.5% [*n* = 13/40], *p* = 0.02) and akinesia (15.0% [*n* = 3/20] versus 50.0% [*n* = 20/40], *p* = 0.008) as reasons compared with patients who stopped driving preoperatively. Other symptoms than listed in the questionnaire were reported as “free-text” more often as reasons to quit driving after surgery compared with before surgery (20.0% [*n* = 4/20] versus 2.5% [*n* = 1/40], *p* = 0.04), namely, “lateral shift of the body” (*n* = 1), “weakness of the legs” (*n* = 1), “car accident” (*n* = 1), and “impulsive and reckless driving” (*n* = 1). The frequency of all other investigated symptoms did not differ significantly in patients who had quit driving either before or after DBS surgery (Figure 1).

### 3.4. Retrospective Assessment of Driving Practice at Time of DBS Surgery

The majority of patients resumed driving shortly after DBS surgery. Three months preoperatively, 66 of the 110 patients (60.0%) were active drivers, but 64.7% of patients expected at that time to be able to drive postoperatively. 50.9% of all patients resumed driving within a postoperative period of 3 months. 12 months after surgery, this proportion increased to 62.4%. One patient had not reached the one-year follow-up period at time of survey.

### 3.5. Influence of Age, Disease Duration, and Motor or Cognitive Impairment on Driving Practice at Time of DBS Surgery

Here we focus on the direct effect of DBS on driving and aim to minimize the influence of disease progression on driving. Therefore, we defined patients as “preoperatively active” drivers, when driving more than 30 min/week within 3 months before surgery and as “postoperatively active” drivers, when driving more than 30 min/week within 1 year after surgery. Other patients were defined as “preoperatively inactive” or “postoperatively inactive” drivers.

H&Y data at clinical “on” state at time of surgery were recorded in 102/110 (92.7%) patients. UPDRS III data of the preoperative levodopa-test were available in the “off” stage in 75/110 (68.2%) and in the “on” stage in 77/110 (70.0%) of patients. MMSE scores were available in 109/110 (99.1%) patients.  Table 2 shows patient characteristics of all drivers and compares characteristics of active and inactive drivers at time of surgery. Furthermore, a comparison of patients who did or did not restart driving is given.

All active drivers at this time drove more than 30 min/week. Age, disease duration, and MMSE score did not differ between pre- or postoperatively active and inactive drivers.

*Preoperatively* active drivers were by trend less severely affected according to H&Y than inactive drivers (*p* = 0.06). Whereas UPDRS III “on” scores were not different, UPDRS III scores in “off” condition were lower (i.e., better) in active than in inactive drivers. Likelihood to drive a car preoperatively decreased by trend with increasing disease severity according to H&Y (*r* = −0.188, *p* = 0.06). Percentages of active drivers were 3.4% in H&Y 1, 37.3% in H&Y 2, 45.8% in H&Y 3, 11.9% in H&Y 4, and 1.7% in H&Y 5.

Patients who drove* postoperatively* had a lower (i.e., better) H&Y score at time of surgery than postoperatively inactive drivers. The UPDRS score was lower in postoperatively active than in postoperatively inactive drivers in “off” but not “on” condition. Likelihood to drive a car postoperatively decreases with increasing disease severity according to H&Y at surgery (*r* = −0.293, *p* = 0.003). Percentages of active drivers were 3.2% in H&Y 1, 38.7% in H&Y 2, 50.0% in H&Y 3, 6.5% in H&Y 4, and 1.6% in H&Y 5.

### 3.6. Effect of DBS on Individual Daily Routine Driving Practice

Within 12 months after DBS surgery, 9.1% (*n* = 10/110) of patients reported resumption and 6.4% (*n* = 7/110) cessation of active driving. Thus, 22.7% (10/44) of patients changed from preoperatively inactive to postoperatively active and 10.6% (7/66) from preoperatively active to postoperatively inactive drivers. Two of these 7 patients stopped driving due to reasons unrelated to their disease. Age, gender, MMSE, and motor impairment according to H&Y and UPDRS III at time of surgery were not significantly different in patients restarting driving after surgery compared to patients without resumption of driving (Table 2). None of the 10 patients restarting driving reported difficulties with orientation, tiredness, or sleep attacks. However, these symptoms were more prevalent in the 34 patients who did not restart driving (*p* = 0.08, *p* = 0.02, and *p* = 0.03).

### 3.7. Predictive Value of the Preoperative Levodopa-Test on Postoperative Driving Behavior

Change of UPDRS motor score in the preoperative levodopa-test was recorded in 75 of 110 (68.2%) patients. Mean motor UPDRS score in the “off” state was 37.7 [11.7] points and in the “on” state 18.4 [7.3] points. Mean improvement was 19.2 [9.3] points, which is 51.8% [17.7] (Table 2). Improvement was not different when comparing postoperatively active and inactive drivers on group (*p* = 0.18) or single subject (*p* = 0.17) levels and so not predictive for the condition of “active driver postoperatively” or switching from “preoperatively inactive” to “postoperative active” driver (*p* = 0.91).

## 4. Discussion

This study aimed to assess daily driving behavior and to reflect surgery-related change of driving practice in PD patients with DBS. We evaluated the current frequency of driving, the influence of patient characteristics, and DBS on present driving behavior and in addition retrospectively the immediate effect of DBS surgery and the predictive value of the preoperative levodopa-test on postoperative driving practice. The study design did not allow the evaluation of driving skills or performance.

A main result of this study is that half of the PD patients with DBS are active drivers. The “population-based” (within the population of DBS patients) study character likely makes this finding transferrable to other PD patients with DBS. The driving-rate of 50% is comparable with the driving-quote of 60% described for treatment-unselected PD patients in another questionnaire survey [7].

In our consecutively investigated PD patients with DBS, the STN was targeted in 93.6% of cases. Therefore our findings represent mainly the situation in the group of PD patients with STN surgery. Due to the small number of target locations other than the STN, the statistical power for subgroup analyses is too low and it is possible that stimulation of VIM or GPi has different implications for driving behavior.

We found active drivers with DBS to be younger and more often males and to have lower disease severity, higher MMSE scores, and shorter duration of DBS compared to inactive drivers. The negative impact of age and cognitive decline on driving is known for PD patients [2, 8] and was recently confirmed for PD patients with DBS tested in a driving-simulator setting [4]. In contrast to disease severity, which has been found to be a risk factor for driving [8], disease duration was estimated to be not predictive of driving performance in PD patients in general [9] and in PD patients with DBS [4]. Accordingly, in the present study, disease duration was not a factor for cessation of car-driving at time of survey or at time around DBS surgery. DBS had influenced self-rated driving ability more often positively (39.4%) than negatively (11.1%), which supports the hypothesis that DBS might improve driving in PD patients [10]. However, driving behavior at time around surgery has been evaluated retrospectively and therefore might be reflected imprecisely.

For the preoperative 3-month period, a higher (i.e., worse) motor UPDRS score in the “off” condition, but not the UPDRS III or H&Y score in the “on” state, was found to be associated with a higher prevalence of inactive drivers. This is in accordance with 2 Class II studies, describing only the motor UPDRS “off” score to be probably predictive of driving performance and 4 Class II and 2 Class III studies describing the H&Y stage to be probably not predictive of driving performance [9].

For the 12-month period postoperatively, a higher motor impairment expressed by higher UPDRS III “off” and H&Y “on” scores at time of surgery was associated with more patients having discontinued driving.

Motor improvement in the preoperative levodopa-test was not predictive of being an active driver within one year postoperatively or of changing from being preoperatively an inactive to becoming postoperatively an active driver. These findings are in line with studies showing an inconsistent and therefore probably not essential influence of motor impairment on driving in PD patients [11–13]. In fact our results support the recent hypothesis that the beneficial effect of STN-DBS on driving in a simulator setting might rather be related to nonmotor aspects of the disease with relevance to driving performance [4, 10].

The postoperative reduction of tremor and akinesia likely explains that both symptoms have only influenced patients before but not after brain surgery in their decision to quit driving. In contrast to patients who did not restart driving after DBS surgery, patients resuming driving did not report orientation problems, tiredness, and sleep attacks at all. Less sleepiness might be related to a postoperative reduction of medication. Positive effects of DBS on orientation could be possible, as recently DBS of the STN has been shown to modulate spatial attention [14]. One patient reported to have quit driving postoperatively due to impulsive and reckless driving, which might be related to stimulation, but also has been described as a dopaminergic side effect [15].

Restarting driving after DBS surgery does not imply automatically improved driving safety due to stimulation. We found 5.5% of patients with DBS who had been involved in a car accident in the last 12 months. However, we did not evaluate accident rates 12 months before and after DBS surgery which might had been more reliable to investigate the direct DBS effect on accident frequency. On the other hand, in several patients DBS had been performed years ago and recall of, for example, minor accidents likely is much more biased than the basic question whether someone did or did not drive within 12 months before or after DBS surgery. Regarding accident rates, there are no directly comparable studies in treatment-unselected drivers with PD. With an approximate estimation of an even distribution of crashes over consecutive years, this crash rate seems to be lower compared with the accident rate of 15.5% in a 2-year follow-up in 106 treatment-unselected PD patients [3] but higher compared to a car crash rate of 15% in 3257 active drivers during a 5-year follow-up study [7].

Noteworthy, car crashes were more frequent in patients considering themselves as safe drivers, which matches findings that patients frequently overestimate their ability to drive [16]. However, we did not evaluate annual mileage of patients and so higher crash rate in drivers feeling safe might be related to a higher mileage compared with drivers feeling unsafe. In contrast to our expectation based on findings of others [11], accident rates were not higher in patients with lower cognitive function at time of survey. This could be related to the low total number of patients with car crashes in our study (*n* = 6).

On the other hand the MMSE is not a comprehensive measurement tool and only gives a coarse picture of the patients' cognitive status. Therefore likely it is not an ideal predictor of driving performance in PD patients [9]. However, our study did not intend to evaluate driving performance but driving behavior and frequency, which probably is not strictly correlated to cognitive capacity. It is known that demented patients often are noncompliant regarding advices not to drive [17].

Of course we routinely do more sensitive cognition scores like the Montreal Cognitive Assessment (MOCA) or the Mattis Dementia Rating Scale or intensive neuropsychological testing in PD patients before DBS surgery. But the preoperative cognitive assessment battery has changed in our center over the last years and not all of the patients in our study were operated on in our center, so we had different preoperative and also different and incomplete postoperative cognitive testing in our patients. In contrast to MOCA or Mattis tests, we perform the much less time consuming MMSE not only preoperatively as screening test but also routinely in the outpatient clinic in almost any follow-up visit of the patients postoperatively. In this study, we only included patients with complete data sets, so we used the MMSE as cognition parameter. Although it lacks sufficient measurement of executive function the MMSE allows a reasonable estimation whether patients are demented or not and whether active and inactive drivers differ regarding their cognition.

Dependency on driving a car in everyday life and reduced QoL without driving were reported more often by active than inactive drivers. Thus, resumption of driving postoperatively in 10 of 44 preoperative inactive drivers might be influenced by these aspects more than by real improvement of driving ability.

Maintenance or regain of driving ability seems to be an important outcome aim for patients undergoing DBS. 96.8% of the postoperatively active and 46.3% of the postoperatively inactive drivers declared to have expected preoperatively to be able to drive after surgery. The latter finding indicates that a notable amount of patients expects DBS to have a positive effect on their driving competence. We suggest to discuss this topic presurgically and if necessary to temper false expectations of the surgical candidates.

An overall benefit from DBS was reported more often in the group of active than of inactive drivers. Active drivers also affirmed more often that they would decide for themselves DBS surgery again. Driving seems to be more important for males than for females. Males more often reported to have expected to be able to drive postoperatively than females, in fact drove more frequently after surgery, and considered themselves more often as safe drivers.

In the present study, the treating physician was aware of PD patients' driving practice in only 30.0% of the cases. In general, the physician has the medical and legal obligation to advise his patients not to drive if they are likely incapable of safe driving [18]. PD patients with DBS probably should not be assessed more critical than other PD patients [4] but it should be considered that neurologists frequently overestimate the capacity to drive in their PD patients [16]. Furthermore, 56 of 110 PD patients drove within 3 months postoperatively. At least the 93 patients, who have been operated on in our center, have been verbally advised before or in the days after implantation of DBS-electrodes that driving is not allowed within 3 months after brain surgery. Despite that, 45 of them drove within 3 months postoperatively. Therefore, adherence to driving ban was low (51.6%).

This study has some limitations. Owing the exploratory nature of the study our findings are tentative and further confirmatory research is thus needed. The retrospective study character with an average completion of the survey 4.1 years after surgery contains a potential risk of selective recall of adverse events such as minor accidents and periods of driving cessation. Also it cannot be excluded that answers were biased because patients were embarrassed to admit driving insufficiency or accidents.

## 5. Conclusion

Like otherwise treated PD patients, patients with DBS frequently drive a car. DBS surgery influences driving behavior and seems to have a positive effect on daily routine driving practice. Disease severity in clinical “off” state at time of brain surgery is a negative predictor for being an active driver postoperatively. Driving after brain surgery is more likely in younger and less motor and cognitive impaired PD patients. The likelihood for driving decreases with duration of brain stimulation. Driving a car after DBS surgery is a relevant aspect for an improving of quality of life, especially for males. Tremor and akinesia appear to be the main driving-relevant motor aspects improved by brain stimulation. The preoperative levodopa-test is not predictive for being or becoming again an active driver after DBS surgery.

## Figures and Tables

**Table 1:** The table shows patient characteristics of drivers at time of survey. Data are shown as means with standard deviation [SD]. Significant different frequencies between active and inactive drivers are labeled with an asterix (*∗*). “H&Y”: Hoehn and Yahr; “QoL”: quality of life.

	Time of survey			

	All (*n* = 110)	Active drivers (*n* = 55)	Inactive drivers (*n* = 55)	*p* value

Age	65.2 [9.4]	63.3 [8.8]	67.0 [9.7]	0.04^*∗*^
Gender (male) (%)	68.2	78.2	58.2	0.02^*∗*^
H&Y stage	3.0 [0.9]	2.6 [0.7]	3.5 [1.0]	<0.001^*∗*^
MMSE (total points)	27.0 [2.9]	28.0 [1.7] (*n* = 54)	26.0 [3.3]	<0.001^*∗*^
MMSE category (%)				
Normal (>26)	71.6	83.3	60.0	0.008^*∗*^
Borderline (24–26)	20.2	14.8	25.5	
Dementia likely (<24)	8.3	1.9	14.5	
Disease duration (years)	16 [6.5]	14.9 [6.4]	17.1 [6.4]	0.08^*∗*^
Driving experience (years)	39.1 [13.3]	41.0 [12.0]	37.0 [14.4]	0.12
Duration of DBS (years)	4.1 [3.2]	3.5 [2.7]	4.7 [3.6]	0.05^*∗*^
Dependency on a car (%)	46.3	70.9	20.8	<0.001^*∗*^
QoL reduced without driving (%)	61.1	81.8	39.6	<0.001^*∗*^
Could rely on another driver (%)	82.7	83.6	81.8	0.80
Total benefit from DBS (%)	92.7	98.2	87.3	0.02^*∗*^
Would do DBS again (%)	90.0	98.2	81.8	0.002^*∗*^

**Table 2:** The table shows patient characteristics of all drivers and compares patient characteristics of active and inactive drivers at time of DBS surgery (within 3 months before and 12 months after surgery). Furthermore, a comparison of patients who did or did not restart driving is given. Data are shown as means with standard deviation [SD]. Significant different frequencies between active and inactive drivers are labeled with an asterix (*∗*).

		Time of DBS surgery			

Within 3 months preoperatively	*N*	All (110)	Active drivers (*n* = 66)	Inactive drivers (*n* = 44)	*p* value

Age (years)	110	61.1 [9.1]	61.2 [8.2]	60.9 [10.3]	0.89
Gender (male) (%)	110	68.2	80.3	50.0	<0.001^*∗*^
H&Y stage (stage)	102	2.8 [0.8]	2.7 [0.8]	3.0 [0.7]	0.06
Disease duration (years)	110	11.9 [5.6]	11.5 [5.4]	12.7 [5.9]	0.29
MMSE (total points)	109	28.5 [1.5]	28.5 [1.6] (*n* = 65)	28.5 [1.3]	0.83
MMSE category (%)	109				
Normal (>26)	96	88.1	86.2	90.9	0.45
Borderline (24–26)	13	11.9	13.8	9.1	
UPDRS III in “ON” (score)	77	18.4 [7.3]	18.0 [6.7]	19.0 [8.1]	0.58
UPDRS III in “OFF” (score)	75	37.7 [11.7]	35.2 [10.3]	41.7 [12.8]	0.02^*∗*^
Improvement in levodopa-test (%)	75	51.8 [17.7]	49.8 [18.0]	54.7 [17.0]	0.23

Within 12 months postoperatively	*N*	All (110)	Active drivers (*n* = 69)	Inactive drivers (*n* = 41)	*p* value

Age (years)	110	61.1 [9.1]	60.5 [8.7]	62.3 [9.6]	0.31
Gender (male) (%)	110	67.9	77.9	51.2	0.004^*∗*^
H&Y stage (stage)	102	2.8 [0.8]	2.6 [0.7]	3.1 [0.8]	0.003^*∗*^
MMSE (total points)	108	28.5 [1.5]	28.6 [1.5] (*n* = 67)	28.3 [1.5] (*n* = 41)	0.37
MMSE category (%)	108				
Normal (>26)	95	88.0	91.0	82.9	0.22
Borderline (24–26)	13	12.0	9.0	17.1	
Disease duration (years)	110	11.9 [5.6]	11.5 [5.8]	12.8 [5.3]	0.25
UPDRS III in “ON” (score)	77	18.4 [7.3]	17.8 [6.8]	19.2 [8.0]	0.41
UPDRS III in “OFF” (score)	75	37.7 [11.7]	34.7 [10.1]	42.1 [12.7]	0.006^*∗*^
Improvement in levodopa-test (%)	75	51.8 [17.7]	49.4 [19.1]	55.1 [15.4]	0.18

Patients restart driving after DBS surgery	*N*	All (110)	Yes [*n* = 10]	No [*n* = 100]	*p* value

Age (years)	110	61.1 [9.1]	56.6 [10.7]	61.5 [8.8]	0.11
Gender (male) (%)	110	68.2	70.0	68.0	0.90
H&Y stage (stage)	102	2.8 [0.8]	2.7 [0.5]	2.8 [0.8]	0.57
MMSE (total points)	109	28.5 [1.5]	28.9 [1.1] (*n* = 10)	28.4 [1.5] (*n* = 99)	0.36
MMSE category (%)	109				
Normal (>26)	96	88.1	100.0	86.9	0.10
Borderline (24–26)	13	11.9	0.0	13.1	
Disease duration (years)	110	11.9 [5.6]	12.8 [8.0]	11.9 [5.4]	0.60
UPDRS III in “ON” (score)	77	18.4 [7.3]	18.3 [7.7]	18.4 [7.3]	0.98
UPDRS III in “OFF” (score)	75	37.7 [11.7]	40.7 [9.7]	37.6 [11.9]	0.54
Improvement in levodopa-test (%)	75	51.8 [17.7]	52.5 [20.3]	51.7 [17.6]	0.91

## Appendix

### A. English Translation of the Original German Version of the Questionnaire

Questions were aimed at giving an overview of daily routine driving practice and impact of DBS operation on driving behavior. Patients were asked to fill out the questionnaire in the waiting room before visiting the physician. Completeness of the questionnaire was checked by the physician at the end of the consultation and potentially misleading or missing answers were clarified.

#### A.1. Dear Patient, Please Answer the Following Questions

Name: …………………
Surname: …………………
Date of birth: …………………
Age: …………………
Date of surgery: …………………
Duration of disease (years): …………………
(1) Do you own a driving license?
  □ Yes
  □ No

*In case you do not own a driving license, you can end the questionnaire here!*

*Nevertheless please return this questionnaire to us!*

(2) How many years have you been driving a car?
…………Years
(3) Do you still drive a car?
    □ Yes
    □ No
  Would you consider yourself as a safe driver?
    □ Yes
    □ No
  Have you been involved into a car accident within the last 12 months?
    □ Yes
    □ No
  Did DBS surgery* increase* your ability to drive?
    □ Yes
    □ No
  Did DBS surgery* decrease* your ability to drive?
    □ Yes
    □ No
  Are you dependent on driving a car (going shopping, going to the doctor etc.)?
    □ Yes
    □ No
  Is/would your quality of life be significantly impaired without the ability to drive a car?
    □ Yes
    □ No
  Can you rely on someone else, who could give you a lift by car as required?
    □ Yes
    □ No
(4) (4a) Did you drive within* 3 months before* DBS surgery?
    □ Yes (More than 30 min/week)
    □ Yes (Less than 30 min/week)
    □ No
  (4b) Did you drive within* 1 year before* DBS surgery?
    □ Yes (More than 30 min/week)
    □ Yes (Less than 30 min/week)
    □ No
  (4c) Did you drive within* 3 months after* DBS surgery?
    □ Yes (More than 30 min/week)
    □ Yes (Less than 30 min/week)
    □ No
  (4d) Did you drive within* 1 year after* DBS surgery?
    □ Yes (More than 30 min/week)
    □ Yes (Less than 30 min/week)
    □ No
(5) Please only answer questions (5a) and (5b), if you have discontinued driving* before DBS surgery*
  (5a) Did you discontinue driving due to Parkinson's disease?
    □ Yes
    □ No
  (5b) If this is the case, what influenced this decision? (multiple answers possible)
  Tremor
    □ Yes
    □ No
  Slowed reactions
    □ Yes
    □ No
  Stiffness/Rigidness
    □ Yes
    □ No
  Dyskinesias
    □ Yes
    □ No
  Orientation problems
    □ Yes
    □ No
  General excessive demand (unable to cope)
    □ Yes
    □ No
  Fatigue
    □ Yes
    □ No
  Sleep attacks (falling asleep at wheel)
    □ Yes
    □ No
  Double vision
    □ Yes
    □ No
  Other impairment of vision
    □ Yes
    □ No
  Impairment of concentration
    □ Yes
    □ No

Others (please specify) …………………

(6) Did you expect to be able to drive again after DBS surgery?
  □ Yes
  □ No
(7) Please only answer questions (7a) and (7b), if you have discontinued driving* after DBS surgery*
  (7a) Did you discontinue driving due to Parkinson's disease?
    □ Yes
    □ No
  (7b) If this is the case, what influenced this decision? (multiple answers possible)
  Tremor
    □ Yes
    □ No
  Slowed reactions  
    □ Yes
    □ No
  Stiffness/Rigidness
    □ Yes
    □ No
  Dyskinesias
    □ Yes
    □ No
  Orientation problems
    □ Yes
    □ No
  General excessive demand (unable to cope)
    □ Yes
    □ No
  Fatigue
    □ Yes
    □ No
  Sleep attacks (falling asleep at wheel)
    □ Yes
    □ No
  Double vision
    □ Yes
    □ No
  Other impairment of vision
    □ Yes
    □ No
  Impairment of concentration
    □ Yes
    □ No

Others (please specify)…………………

(8) Overall, did you benefit from DBS surgery?
  □ Yes
  □ No
(9) Assuming the result remained the same, would you decide on DBS surgery again?
  □ Yes
  □ No

*Thank you very much for participating in this study*

#### A.2. The Subsequent Part Is Filled Out by the Physician

Name: …………………
Surname: …………………
Date of birth: …………………
Questionnaire complete?
  □ Yes
  □ No
Target of DBS surgery?
  □ STN
  □ GPI
  □ VIM unilat.
  □ VIM bilat.
Subtype of Parkinson's Disease?
  □ Equivalent
  □ akinetic-rigid
  □ tremor-dominant
  □ un-known
Present Hoehn & Yahr stage
  □ 1-2
  □ 3
  □ 4-5
  □ un-known
Center of DBS surgery UKE Hamburg?
  □ Yes
  □ No
Microelectrode recordings during surgery?
  □ Yes
  □ No
Did you know, whether the patient at present drives a car?
  □ Yes
  □ No

Signature Physician

…………………

Place, Date

…………………
